# Investigation into electro mechanical behaviour of MEMS wing shaped dielectric capacitive pressure sensor conforming to set boundary conditions and sizing tolerances

**DOI:** 10.1038/s41598-025-08654-3

**Published:** 2025-07-07

**Authors:** Gajula Rukshana Bi, Sumit Kumar Jindal, Suraj Prakash Sahoo

**Affiliations:** School of Electronics Engineering, Vellore Institute of Technology, Vellore, Tamil Nadu 632014 India

**Keywords:** MEMS, Touch mode capacitive pressure sensor, Circular diaphragm, Wing-shaped dielectric, MATLAB, COMSOL, Electrical and electronic engineering, Mechanical engineering

## Abstract

Micro Electro Mechanical Systems (MEMS) based Capacitive Pressure Sensors (CPS) have shown their versatility and reliability in numerous applications. In this work, an innovative approach to optimize sensor performance through the integration of a novel wing-like mechanism is proposed. Unlike traditional CPS this mechanism employs two insulating layers attached to the upper layer of the substrate wing-like structure, which opens when pressure is applied, ultimately leading to the formation of a touch point. By decreasing the distance, the diaphragm must deflect before making contact, making it more responsive to applied pressure the sensor’s sensitivity is greatly increased. Key performance metrics including capacitance variations, capacitive sensitivity and the deflection of diaphragm using small deflection theory have been analytically evaluated. To verify the analytical calculations of capacitance, capacitive sensitivity, and mechanical sensitivity MATLAB is used, while COMSOL Multiphysics is utilized to validate the diaphragm’s deflection.

## Introduction

The advancement of microscale fabrication technology, particularly Micro-Electro Mechanical Systems (MEMS), has significantly broadened the operating range of pressure sensors, enabling them to measure pressures from extremely low to exceptionally high levels^1^. Owing to their vital role in practical applications, significant efforts have been dedicated to enhancing the modeling and design of these sensors. Healthcare, industrial automation, automotive, and aerospace are just a few of the industries that have used them^2–5^. Pressure sensors are categorized based on the principle of deformation of a sensing device, usually a diaphragm, in response to applied pressure as capacitive, resonant, optical, piezoresistive, or piezoelectric sensors^6^. Piezoresistive pressure sensors, which use a silicon diaphragm embedded with piezo resistors configured in a Wheatstone bridge, operate based on the piezoresistive effect, where electrical resistance changes with respect to mechanical stress. As pressure is applied, the diaphragm deforms and produces stress and strain, which alter the band structure and carrier mobility. While the applied pressure varies, the resistance changes proportionally and is then transformed into a corresponding electrical signal^7^. Resonant pressure sensors are sensors that use a shift in resonant frequency to measure pressure. Because of their excellent accuracy and stability, they are well suited for high-precision and long-term reliability applications. However, their use in cost-sensitive consumer electronics is restricted because of their complex design and high production costs^8,9^. By sensing change in optical characteristics like intensity, phase, or wavelength caused on by the deformation of a flexible diaphragm under applied pressure, an optical pressure sensor may determine pressure. Precise pressure readings are obtained by directing light from a laser or LED over an optical fiber to the sensor region, where differences in the transmitted or reflected signal brought on by diaphragm movement are analyzed^10,11^.

Applications requiring high sensitivity and high pressure and temperature operating conditions are ideally suited for MEMS capacitive pressure sensors (CPS). They also provide improved stability under different thermal conditions and lower power usage^12,13^. To increase performance, a variability of designs has been proposed, including comb, push–pull, and touch mode configurations. A novel design with a spiral comb electrode has been introduced to increase electrode overlap, thereby improving sensitivity and achieving linearity exceeding 0.99, while minimizing reliance on electrode spacing. This design aims to overcome the high nonlinearity and limited sensitivity of conventional CPS. Over a pressure range of 0–30 kPa, a capacitive sensitivity of 1.10 aF/Pa and a mechanical sensitivity of 1.5 × 10^−4^ m/Pa are observed, with a low nonlinearity of 3.63%. This enhancement is validated experimentally, allowing it to be used for high-sensitivity pressure sensing^14^. Performance increases were notable with the touch mode structure^15–17^.

More attention was paid to Touch Mode Capacitive Pressure Sensors (TMCPS) in the late 1990s, and in 1997 a prototype TMCPS was formed using bonding techniques. The sensor’s two electrodes are made up of a fixed substrate and a flexible diaphragm. CPS measures changes in capacitance induced on by diaphragm deformation to determine pressure. Low pressures cause the diaphragm to deflect but not come into touch with the substrate, allowing the sensor to operate normally. The diaphragm deflects further and comes into contact with the substrate as the pressure rises, which causes the sensor to go into touch mode. This mode provides a near-linear output response^19^ and a wider pressure range than traditional mode. Consequently, this concept has led to the Single Touch Mode Capacitive Pressure Sensor (STMCPS) with a circular diaphragm^20^. The development of the Double Touch Mode Capacitive Pressure Sensor (DTMCPS) addressed the issues of low linearity and early saturation in STMCPS. Compared to conventional STMCPS designs, adding an etched notch at the substrate’s base improves linearity and sensitivity^21^. The DSTMCPS is a result of a major improvement in sensor design achieved by placing two TMCPS on either side of a single substrate^22^. According to simulation results, the linear operating range of the CPS structure with a linkage film (CPSSLF)^23^ is about 2.2 times greater than that of traditional TMCPS designs. Numerous CPS designs and material combinations have been found to improve durability, increase sensitivity, and expand the operational range.

Enhancing the contact area between the diaphragm and the substrate of a TMCPS design will eventually improve the capacitance, sensitivity, and linearity^24^. A novel wing-like mechanism is introduced to increase these metrics by integrating two insulating layers that differ from conventional approaches. One insulating layer covers the diaphragm and the other consists of insulating wings that extend into the cavity and are attached to the substrate, this mechanism allows the diaphragm to deflect and the insulating wings to open under applied pressure. This operation increases the contact area and enhances sensitivity. Furthermore, the study aims to validate the superior sensitivity of this innovative sensor structure compared to its traditional contact mode counterpart by deriving deflection, capacitance and capacitive sensitivity and analysing with MATLAB. Additionally, to validate the diaphragm’s deflection COMSOL Multiphysics is employed.

## Proposed wing like structure and its electro mechanical dynamics

**Fig. 1:**
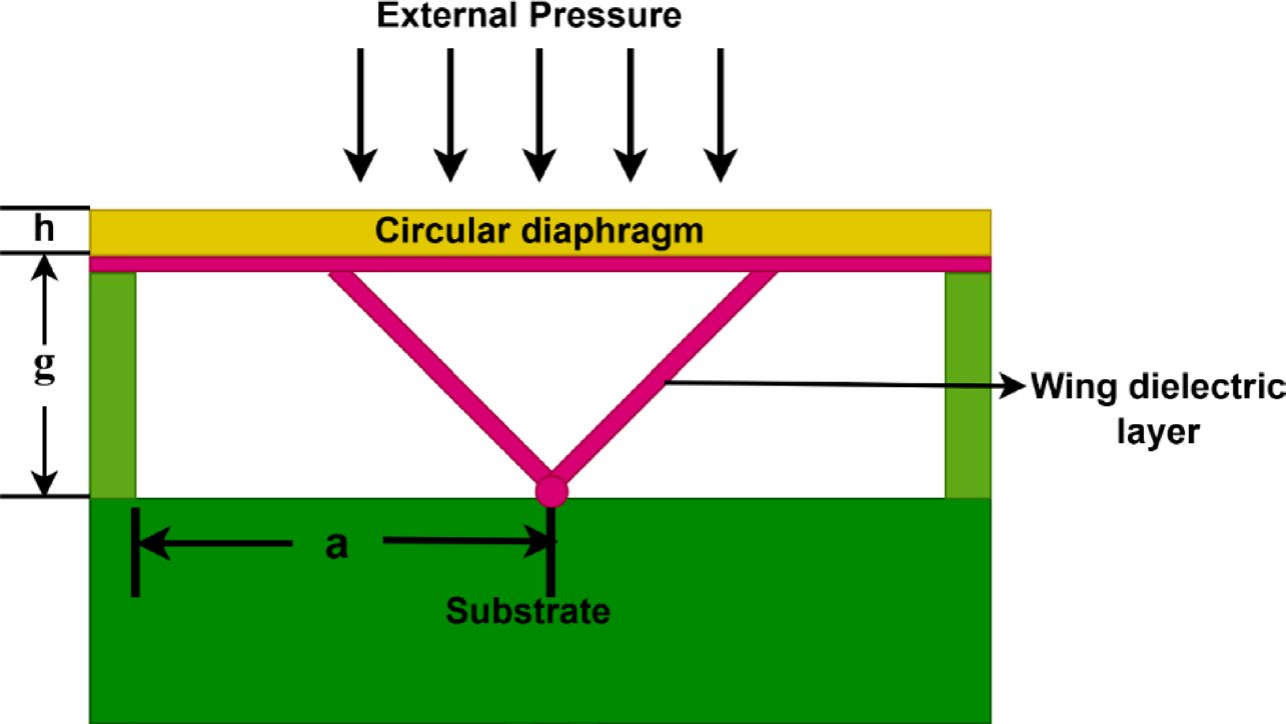
Non-Touching Configuration of the modified capacitive pressure sensor.

Figure 1 illustrates the initial configuration of the sensor, operating in normal operation. The proposed sensor operates by means of a capacitive sensing mechanism, in which a change in capacitance is induced by external pressure. In this configuration, the substrate functions as the bottom, fixed electrode and the diaphragm as the top, flexible electrode, forming the two conducting plates of a capacitor. The structure is composed of two symmetric wing-shaped dielectric structures attached to a central anchor beneath a circular diaphragm that is placed above a Si substrate. The diaphragm deflects downward toward the substrate when pressure is applied, shortening the distance between the conductors and increasing the capacitance. The diaphragm’s deformation principle in sensors relies on both small and large deflection theories. Sensor linearity is improved with small-deflection deformation but decreases with large deflection deformation^15^. The circular diaphragm radius *R* is 180 μm and thickness *h* is 10 μm. A substrate with dimensions of 400 μm × 400 μm serves as a rigid, fixed plate that provides a stable base for the sensor, remaining unaffected by pressure changes.

Silicon (Si) is chosen for the diaphragm and substrate to ensure reliable pressure sensing^25^, owing to its well-established processing technology, thermal stability, favorable mechanical properties, compatibility with integrated electronics, and cost-effectiveness. The two symmetrically positioned slanted dielectric elements between the diaphragm and the center anchor point on the substrate are referred to as the wing structures in Fig. 1. A dielectric material, Silicon Nitride (Si_3_N_4_), is used to form these wings. As opposed to the diaphragm, which is suspended, the dielectric wings are fixed to the substrate. Each wing in this design has dimensions of around 0.5 μm for thickness, 3.6 μm for slant length, 2.99 μm for horizontal base width, and 2 μm for vertical height. This results in a slant angle of about 33.8° with respect to the substrate surface. These values help define a narrow vertical gap while maximizing the effective touch area, thereby enhancing capacitive sensitivity.

Si_3_N_4_ is used due to its exceptional insulating properties, with a resistivity of 10^14^ Ω·cm, which is significantly higher than that of Silicon Dioxide (SiO_2_). The intersection or central point where the two slanted wing-shaped dielectric structures meet at the bottom of the cavity marks the tip. The vertical distance from this tip on the substrate to the diaphragm forms the narrowest gap in the structure and is defined as the gap depth *g*, which is 2 μm in this design. The proposed sensor design parameters are shown in Table 1.

**Table 1:** Design parameters of the proposed sensor.

Parameters	Design Value
Young’s Modulus	$170\times {10}^{9} \text{N}/{\text{m}}^{2}$
Radius of diaphragm (*a*)	180 μm
Thickness of the diaphragm (*h*)	10 μm
Gap Depth (*g*)	2 μm
Thickness of the insulating layer (*t*)	0.5 μm
Wings Slant length (l)	3.6 μm
Slant angle	33.8°
Poisson’s ratio for silicon ($\boldsymbol{\vartheta }$)	0.28
Normal mode pressure range	0–0.28 MPa
Touch mode pressure range	0.28–1.75 MPa
Saturation mode pressure range	Beyond 1.75 MPa

### Functioning of wing-like structure

**Fig. 2:**
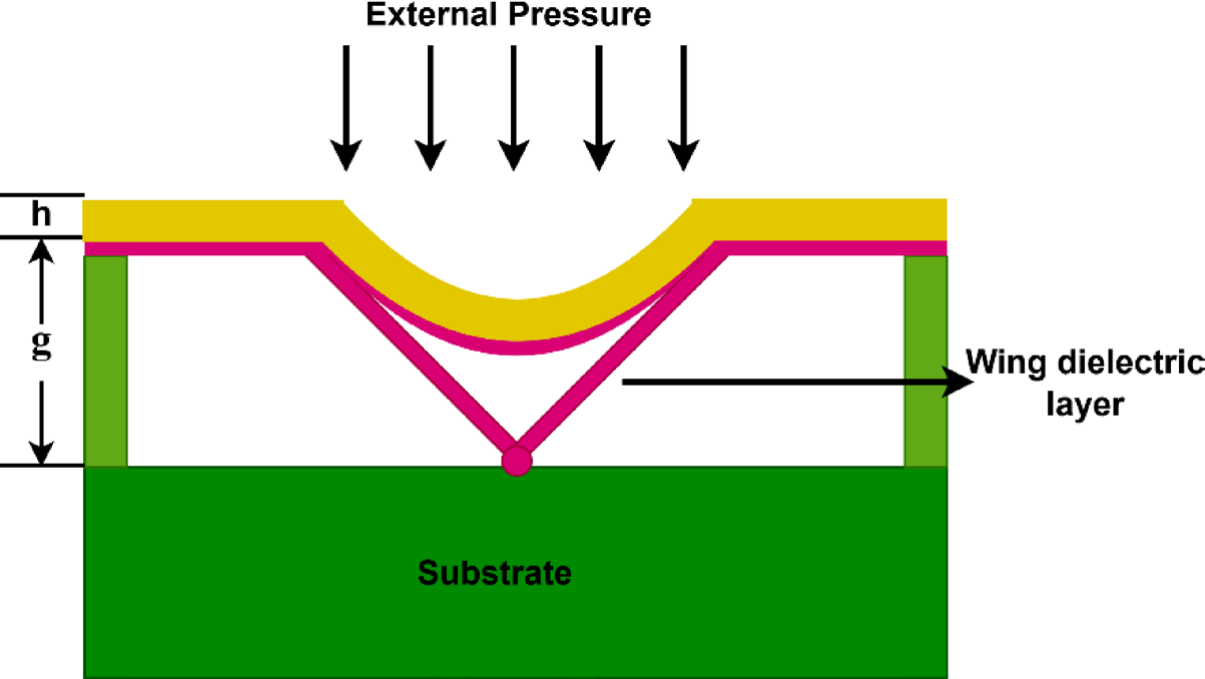
Partial Touch Mode of the modified capacitive pressure sensor.

**Fig. 3:**
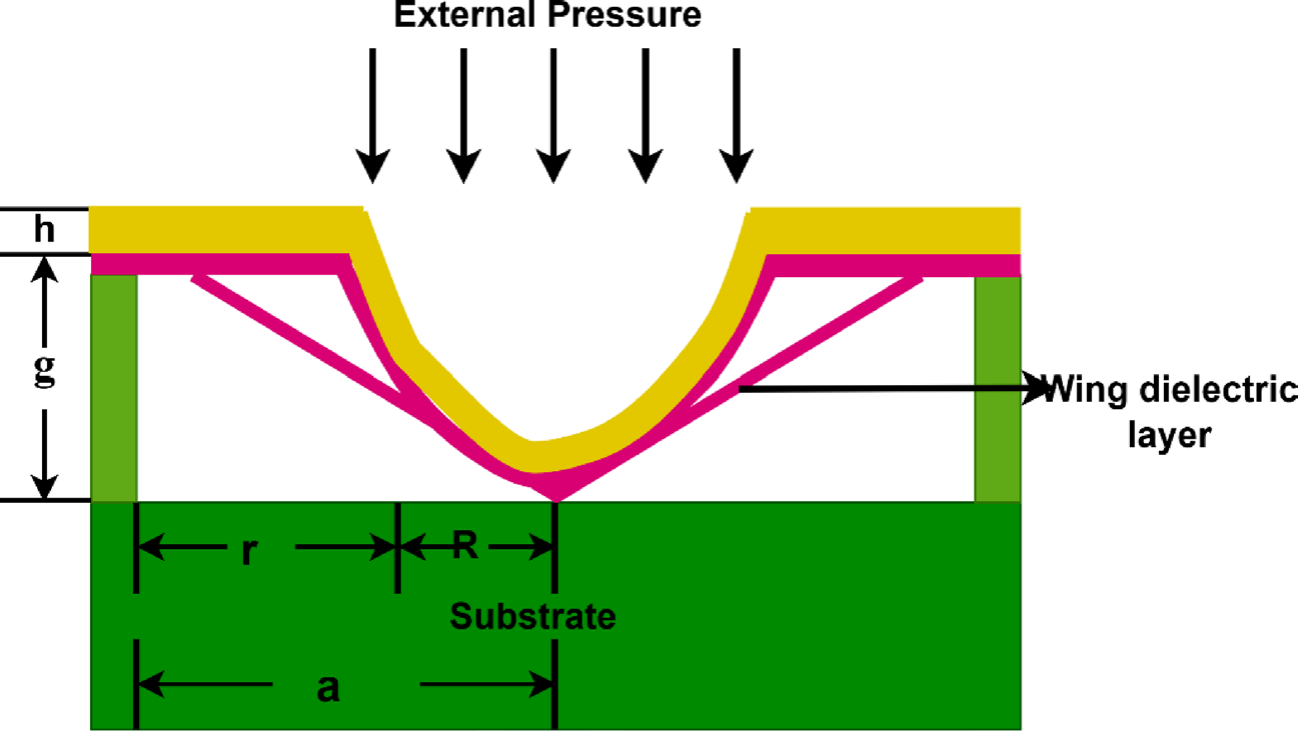
Cross sectional view of touch mode capacitance in CPS.

There are three main modes of operation for the capacitive touch mode pressure sensor: normal, touch, and saturation. Applying pressure causes the diaphragm to slightly deflect toward the substrate, doesn’t makes contact between the two. At this point, the sensor functions in normal mode, which causes an increase in capacitance as a result of the gap decreasing. Further increasing the pressure, the diaphragm deflects more towards the substrate. At this stage the dielectric wings open slightly and the diaphragm contacts the wings dielectric layer on the substrate, causing the sensor to go into touch mode as shown in Fig. 2. Further increase in the pressure makes the diaphragm deflection more and the dielectric wings open more increasing the contact area and also decreasing the gap depth. The capacitance *C* between the diaphragm and the substrate is inversely proportional to the gap *g* between them. As the diaphragm deflects and the gap *g* decreases, the capacitance *C* increases according to the Eq. (1).

1 $$C= \frac{{\varepsilon }_{0 }{\varepsilon }_{r} A}{g}$$

where ${\varepsilon }_{0}$ denotes the permittivity of free space, ${\varepsilon }_{r}$ specifies the relative permittivity of the dielectric material, and the area of the diaphragm is denoted by *A*. In contrast to traditional capacitive pressure sensors, this mode improves linearity by increasing the capacitance more linearly with pressure as a result of the contact area development^18^. Figure 3 presents the sensor in complete touch mode. At this stage, the wing-like mechanism is fully engaged, and the diaphragm is in complete touch mode with the substrate below. In this configuration, the sensor has maximum touch area with the substrate and achieves increased capacitance according to Eq. (1). This results in enhanced capacitive sensitivity. After this the sensor enters into saturation region. The saturation region is the point at which the contact area saturates that is, when the diaphragm can no longer extend the contact region or deform it significantly under high enough pressure. The saturation mode indicates the maximum limit of the sensor’s useful operating range when the capacitance approaches saturation or just slightly increases with added pressure.

## Mathematical Evaluation using allowable Boundary Thresholds

In this section the relationship between applied pressure and capacitance, based on the contact formed between the substrate and the diaphragm as shown in Fig. 3, is derived mathematically. The following Eq. (2) gives the total capacitance.

2 $${C}_{Tm}= {C}_{T}+ {C}_{ut}$$

where, ${C}_{Tm}$ is Total capacitance, ${C}_{T}$ is Touched capacitance, and ${C}_{ut}$ is Untouched capacitance due to radial motion.

### Touched capacitance calculation

In touch mode, the capacitance of the sensor increases due to the deformation or direct contact of the dielectric layer, which reduces the effective separation between the electrodes and alters the contact area. When an external pressure is applied to the sensor, the resulting capacitance can be determined using Eq. (3), which is obtained through the application of the Gauss numerical integration method^26^.

3 $$C={\int }_{0}^{2\pi }{\int }_{0}^{a}\frac{{\varepsilon }_{0}{\varepsilon }_{a}{\varepsilon }_{i} r dr d\theta }{{\varepsilon }_{a}t+{\varepsilon }_{i}\left(g-\omega \left(r\right)\right)}$$

where ${\varepsilon }_{0}$ denotes permittivity of vaccum, ${\varepsilon }_{a}$ and ${\varepsilon }_{i}$ are dielectric constants of air and insulator material.* r* specifies Gaussian point in the range from 0 to *a* and *g* is the distance between diaphragm and substrate. $\omega \left(r\right)$ represents the deflection of the diaphragm as indicated in Eq. (4).

4 $$\omega \left(r\right)=\frac{P({s}^{2}-{r}^{2}{)}^{2}}{64D}$$

where *P, s,* and *D* are differential pressure, radius of the circular diaphragm, and flexural rigidity of the material respectively. Equation (5) specifies the flexural rigidity.

5 $$D=\frac{E{h}^{3}}{12(1-{v}^{2})}$$

where E denotes Young’s Modulus and $\boldsymbol{\vartheta }$ specifies material’s poisons ratio.

On substituting the Eqs. (4) and (5) in Eq. (3) with touched region boundaries and simplifying them yields touched capacitance, ${\text{C}}_{\text{T}}$. i.e.,

6 $${C}_{T}=\frac{{\varepsilon }_{0}{\varepsilon }_{i}\cdot 2\pi }{t}*\underset{0}{\overset{R}{\int }}{r}_{1}{dr}_{1}$$

7 $${\text{C}}_{\text{T}}=\frac{\uppi {\upvarepsilon }_{0}{\upvarepsilon }_{\text{i}}{\text{R}}^{2}}{\text{t}}$$

where *r*_*1*_ is the gaussian point in the range *0* to *R*, which is used to calculate touched capacitance.

### Untouched capacitance calculation

The integral ${C}_{ut}$ represents the capacitance due to radial motion. It is integral to understanding how capacitance changes in response to a complex combination of parameters. It is given by Eq. (8).

8 $$C_{ut} = \frac{{2\pi \varepsilon_{0} \sin \theta }}{\theta }\mathop \int \limits_{0}^{r} \frac{{r_{2} dr_{2} }}{d}$$

where *r*_*2*_ is the gaussian point in the range *0* to *r* which is used to calculate untouched capacitance.Since, $d=g-\omega \left(r\right)$, and $\omega \left(r\right)={\omega }_{0}{\left(1-\frac{{r}^{2}}{{\left({a}^{\prime}\right)}^{2}}\right)}^{2}$where ${a}^{\prime}= \sqrt{{a}^{2}+{g}^{2}}$

On substituting the terms, d, $\omega \left(r\right)$ in Eq. (8) yields untouched capacitance as in Eq. (9).

9 $${C}_{ut}=2\pi {\varepsilon }_{0}\underset{0}{\overset{r}{\int }}\frac{rdr}{g-\omega \left(r\right)}$$

$${C}_{ut}=\frac{2\pi {\varepsilon }_{0}\mathit{sin}\theta }{\theta }\underset{0}{\overset{r}{\int }}\frac{rdr}{g-\frac{P({{a}^{\prime})}^{4}}{64D}{\left(1-\frac{{r}^{2}}{({{a}^{\prime})}^{2}}\right)}^{2}}=2\pi {\varepsilon }_{0}\underset{0}{\overset{r}{\int }}\frac{rdr}{g-{\omega }_{0}\cdot {\left(\frac{({{a}^{\prime})}^{2}-{r}^{2}}{({{a}^{\prime})}^{2}}\right)}^{2}}$$

Let $({{a}^{\prime})}^{2}-{r}^{2}=z$ and differentiating with respect to *r* and applying modified limits to the above equation.

$${C}_{ut}=2\pi {\varepsilon }_{0}\underset{{\left({a}^{\prime}\right)}^{2}}{\overset{{\left({a}^{\prime}\right)}^{2}-{r}^{2}}{\int }}\frac{\frac{-dz}{2}}{g-{\omega }_{0}{\left(\frac{z}{{(a^{\prime})}^{2}}\right)}^{2}} =\frac{\pi {\varepsilon }_{0}}{g}\underset{{\left({a}^{\prime}\right)}^{2}-{r}^{2}}{\overset{{\left({a}^{\prime}\right)}^{2}}{\int }}\frac{-dz}{1-\frac{{\omega }_{0}}{g}{\left(\frac{z}{{\left({a}^{\prime}\right)}^{2}}\right)}^{2}}$$

$${C}_{ut}=\frac{\pi {\varepsilon }_{0}}{g}\underset{{\left({a}^{\prime}\right)}^{2}-{r}^{2}}{\overset{{\left({a}^{\prime}\right)}^{2}}{\int }}\frac{-dz}{1-{\left(\sqrt{\frac{{\omega }_{0}}{g}}*\frac{z}{{(a^{\prime})}^{2}}\right)}^{2}}= \frac{\pi {\varepsilon }_{0}}{g}*\sqrt{\frac{g}{{\omega }_{0}}}*{\left({a}^{\prime}\right)}^{2}*{tanh}^{-1}{\left(\frac{z}{{\sqrt{\frac{g}{{\omega }_{0}}}*\left({a}^{\prime}\right)}^{2}}\right)}_{{\left({a}^{\prime}\right)}^{2}- {r}^{2}}^{{\left({a}^{\prime}\right)}^{2}}$$

$${C}_{ut}= \frac{\pi {\varepsilon }_{0}}{g}*\sqrt{\frac{64Dg}{P{\left({a}^{\prime}\right)}^{4}}}*{\left({a}^{\prime}\right)}^{2}*\left[{\text{tanh}}^{-1}\left(\sqrt{\frac{{\omega }_{0}}{g}}*\frac{{\left({a}^{\prime}\right)}^{2}}{{\left({a}^{\prime}\right)}^{2}}\right)-{\text{tanh}}^{-1}\left(\sqrt{\frac{{\omega }_{0}}{g}}*\frac{{\left({a}^{\prime}\right)}^{2}-{r}^{2}}{{\left({a}^{\prime}\right)}^{2}}\right)\right]$$

$${C}_{ut}=8\pi {\varepsilon }_{0}\sqrt{\frac{D}{Pg}} *\left[{\text{tanh}}^{-1}\left(\sqrt{\frac{{\omega }_{0}}{g}}\right) -{\text{tanh}}^{-1}\left(\sqrt{\frac{{\omega }_{0}}{g}}\frac{{\left({a}^{\prime}\right)}^{2}-{r}^{2}}{{\left({a}^{\prime}\right)}^{2}}\right)\right]$$

10 $${C}_{ut}=2\pi {\varepsilon }_{0}\sqrt{\frac{D}{Pg}} *\left[{\text{tanh}}^{-1}\left(\frac{{\left({a}^{\prime}\right)}^{2}}{8} *\sqrt{\frac{P}{Dg}} \right)-{\text{tanh}}^{-1}\left(\frac{{\left({a}^{\prime}\right)}^{2}}{8} *\sqrt{\frac{P}{Dg}} * \frac{{\left({a}^{\prime}\right)}^{2}-{r}^{2}}{{\left({a}^{\prime}\right)}^{2}}\right)\right]$$

Since, ${C}_{Tm}= {C}_{T}+{C}_{ut}$, the total capacitance,

11 $$\therefore {C}_{Tm}=\frac{\pi {\varepsilon }_{0}{\varepsilon }_{i}{R}^{2}}{t}+ 2\pi {\varepsilon }_{0}\sqrt{\frac{D}{Pg}} *\left[{\text{tanh}}^{-1}\left(\frac{{\left({a}^{\prime}\right)}^{2}}{8} *\sqrt{\frac{P}{Dg}} \right)-{\text{tanh}}^{-1}\left(\frac{{\left({a}^{\prime}\right)}^{2}}{8} *\sqrt{\frac{P}{Dg}} * \frac{{\left({a}^{\prime}\right)}^{2}-{r}^{2}}{{\left({a}^{\prime}\right)}^{2}}\right)\right]$$

### Capacitive sensitivity calculation (S) *(study of C-P relationship)*

Capacitive sensitivity is a key performance metric for evaluating the responsiveness of touch sensors. It is the rate of change of capacitance with respect to applied pressure^26^.

12 $$s=\frac{{\text{dC}}_{\text{Tm}}}{\text{dP}}$$

### Mechanical sensitivity (***s***_***mech***_)- ***(Validation of diaphragm thickness under study)***

In touch mode capacitive pressure sensors (TMCPS), mechanical sensitivity is a key factor that quantifies the diaphragm’s deflection in response to applied pressure, influencing the sensor’s capacitance and overall performance. It is defined as the ratio of the diaphragm’s deflection to the applied pressure.

13 $${s}_{mech}=\frac{w}{p}$$

By simplification,

14 $${s}_{mech}=\frac{0.1875P{R}^{4}(1-{v}^{2})}{E{h}^{3}}$$

From Eq. (14), the mechanical sensitivity of the sensor depends on the fourth power of the diaphragm’s radius and the cube of its thickness. This indicates that increasing the diaphragm’s radius or decreasing its thickness will enhance sensitivity, such changes may reduce the structural integrity of the sensor and limit its operational range. So, it’s essential to choose the right diaphragm dimensions and material properties for an optimal balance between sensitivity and mechanical robustness.

## Findings and interpretations

This work assesses the key metrics of reported MEMS wing shaped TMCPS design through analytical modelling and numerical simulations using MATLAB. The metrics include diaphragm deflection, diaphragm thickness, touch area, capacitance, and capacitive sensitivity for the wing shaped structure of MEMS TMCPS. The design is modelled in COMSOL Multiphysics to validate the diaphragm deflection under applied pressure, ensuring that the mechanical response of the design aligns with theoretical expectations and performance requirements for the model under study.

### Deflection of the diaphragm in response to applied pressure- *(in accordance with small deflection theory of shells and plates)*

**Fig. 4:**
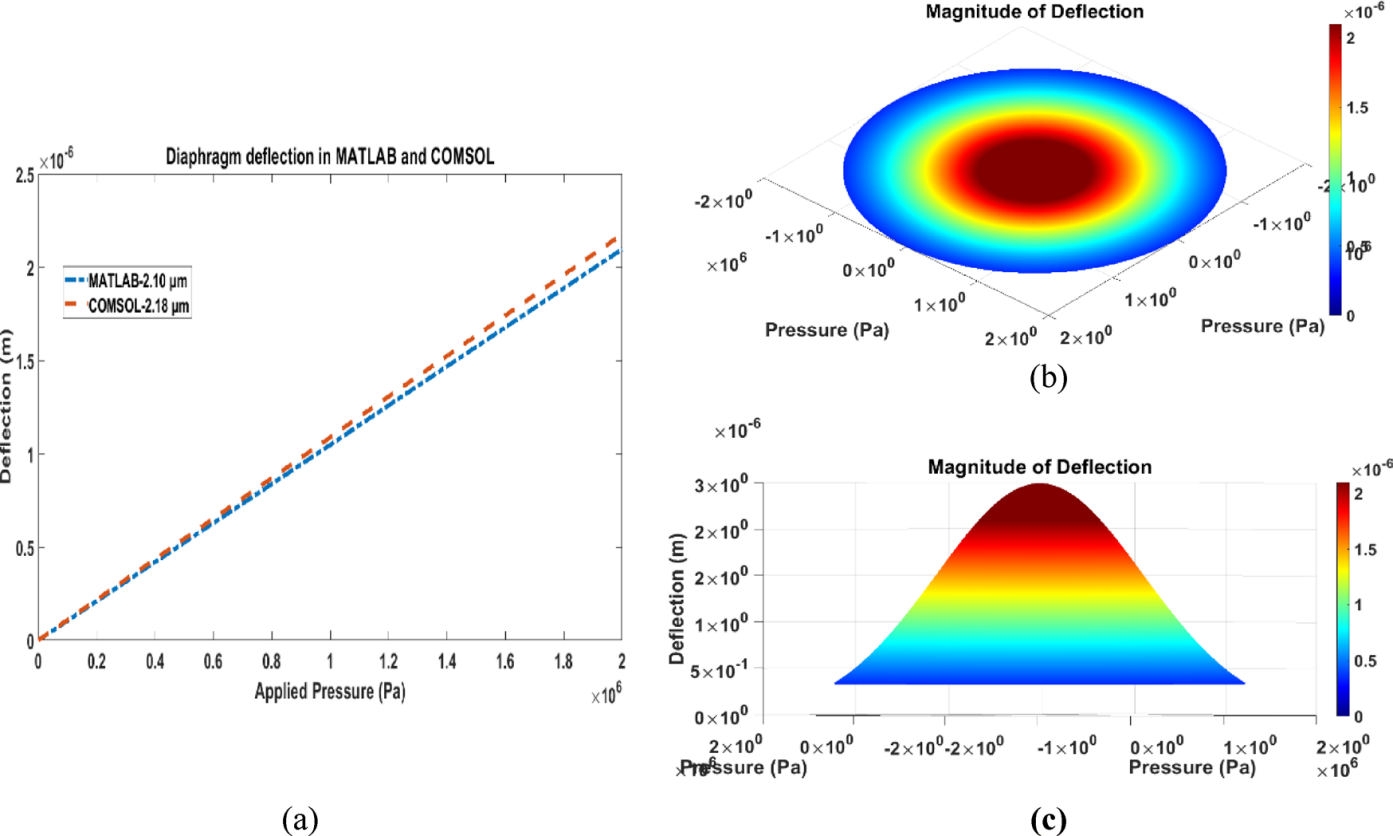
(**a**) Comparison of diaphragm deflection in MATLAB and COMSOL (**b**) Deflection in MATLAB (**c**) 3D view of the diaphragm deflection in MATLAB.

The diaphragm deflection as a function of applied pressure in the range of 0–2 MPa is illustrated in Fig. 4. It is evident that the central deflection of the diaphragm increases proportionally with increasing pressure, exhibiting a linear characteristic. This linear behaviour within the specified range indicates consistent mechanical performance of the sensor structure, which is crucial for predictable and reliable operation. Figure 4a compares the diaphragm deflection obtained from MATLAB and COMSOL simulations. The deflection profile is shown in Fig. 4b, while the 3D view of the diaphragm deflection generated in MATLAB is presented in Fig. 4c.

**Fig. 5:**
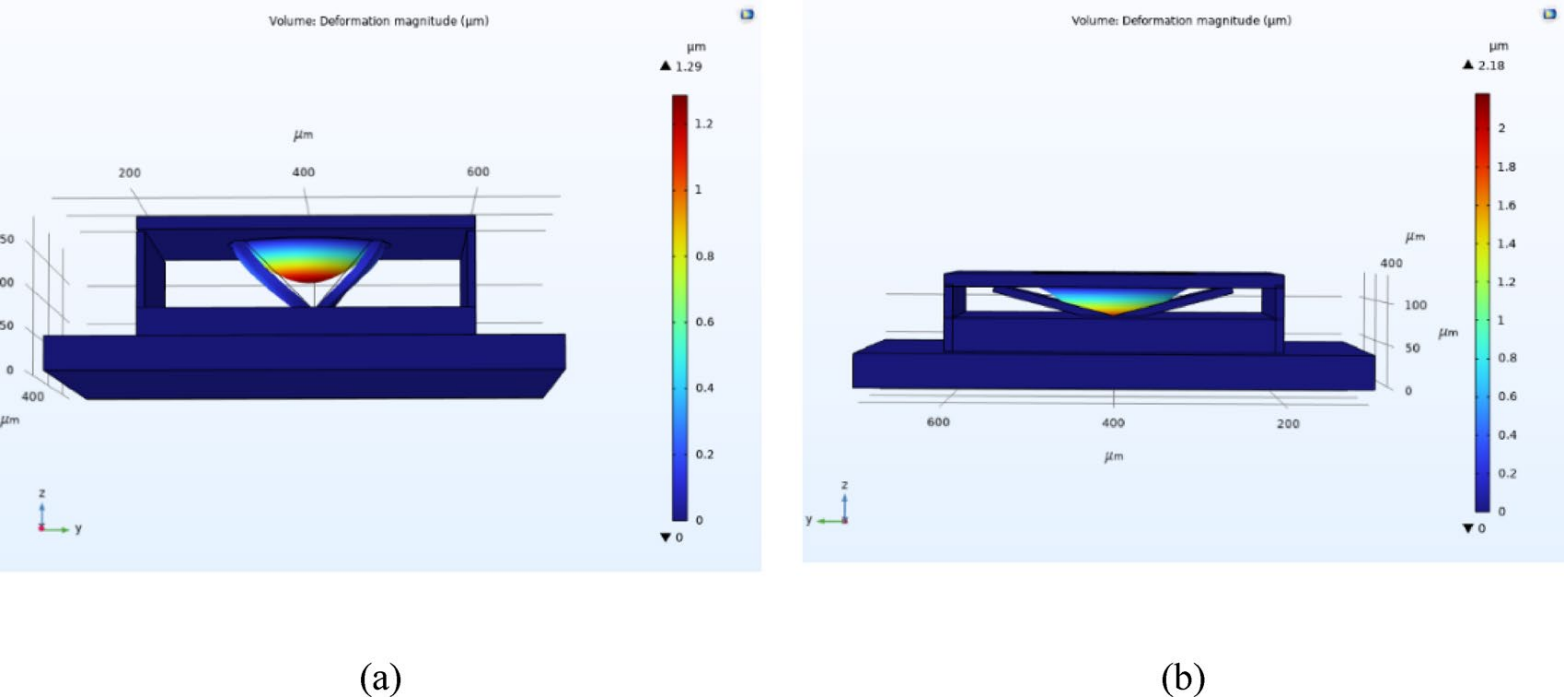
(**a**) Single-touch mode modelling of proposed sensor in COMSOL, (**b**) Sensor response in complete-touch mode.

To validate the diaphragm deflection, the proposed sensor was modelled and simulated in COMSOL Multiphysics. The diaphragm geometry, material properties, and boundary conditions were implemented to closely replicate the conditions assumed in the analytical model. Under an applied pressure of 2 MPa, the maximum diaphragm deflection obtained from the COMSOL simulation was 2.18 µm, while the corresponding deflection calculated using MATLAB was 2.10 µm. The percentage difference between the two results is approximately 3.67%, indicating agreement between the numerical and analytical approaches. Furthermore, since the maximum deflection remains smaller than the diaphragm thickness, the assumptions of small deflection theory are satisfied^26^. The simulated sensor design and deformation results for both single-touch and complete-touch modes are illustrated in Fig. 5.

### Pressure dependent variation in contact area *(enhanced near-linear region)*

**Fig. 6:**
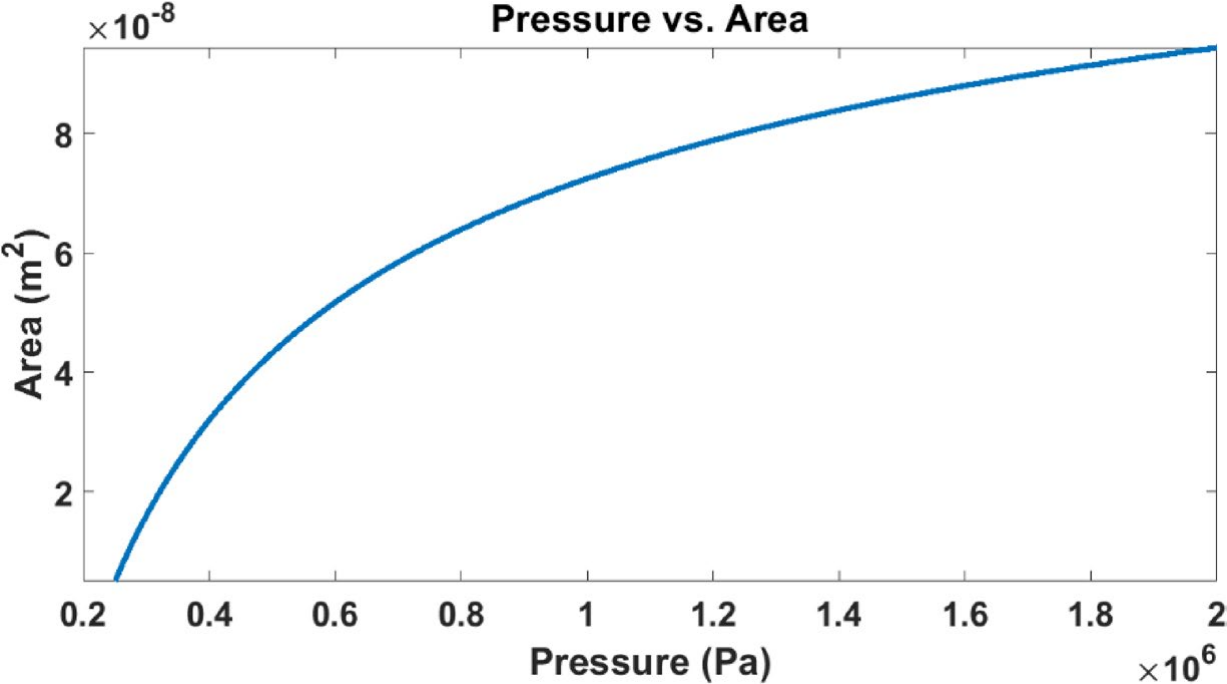
Touch area variation with pressure.

The relationship between touch area and applied pressure in TMCPS is characterized by an initial nonlinear region, followed by a near-linear behaviour. At lower pressures, as the diaphragm approaches the substrate, the capacitance changes nonlinearly due to the varying gap between the electrodes and sensor is in the transition region. Once contact is established, the touch area increases almost proportionally with pressure, resulting in a nearly linear capacitance-pressure relationship as shown in Fig. 6. This linearity enhances sensor performance by providing predictable and stable output within a specific pressure range.

### Capacitance response to applied pressure (the C-P Curve)

**Fig. 7:**
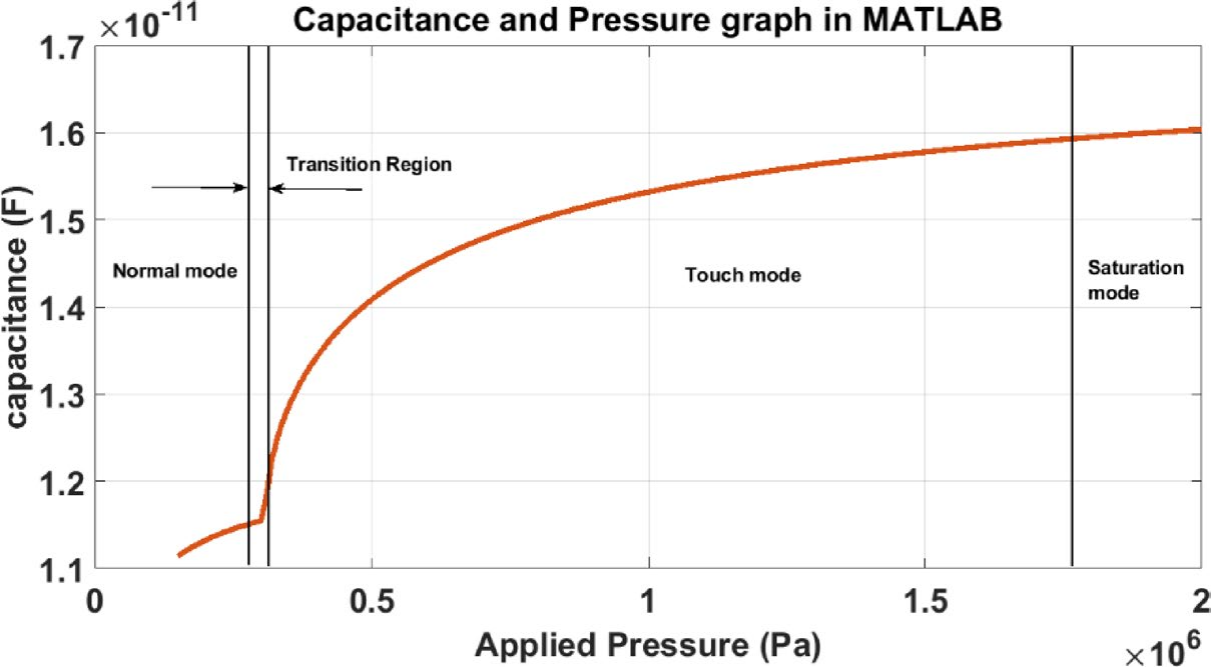
Variation of capacitance with pressure in MATLAB.

Figure 7 illustrates the variation of capacitance with applied pressure, as simulated in MATLAB for a touch mode capacitive pressure sensor. The graph distinctly outlines three major operational regions normal mode (0–0.28 MPa), transition region (0.28–0.3 MPa), and touch mode (0.3–1.75 MPa) followed by a saturation mode (beyond 1.75 MPa). In the normal mode, the diaphragm deforms without making physical contact with the substrate, leading to a gradual increase in capacitance due to the narrowing electrode gap. As pressure increases into the transition region, the diaphragm begins to make initial contact with the substrate, causing a rapid and nonlinear rise in capacitance due to the sharp change in dielectric configuration. This is followed by the touch mode, where the diaphragm remains partially in contact with the substrate and the capacitance continues to increase steadily with pressure as the contact area expands. Finally, in the saturation mode, the diaphragm makes near-complete contact with the substrate, resulting in minimal additional capacitance change despite increased pressure.

### Pressure induced capacitive sensitivity analysis (Investigation of key performance parameter)

**Fig. 8:**
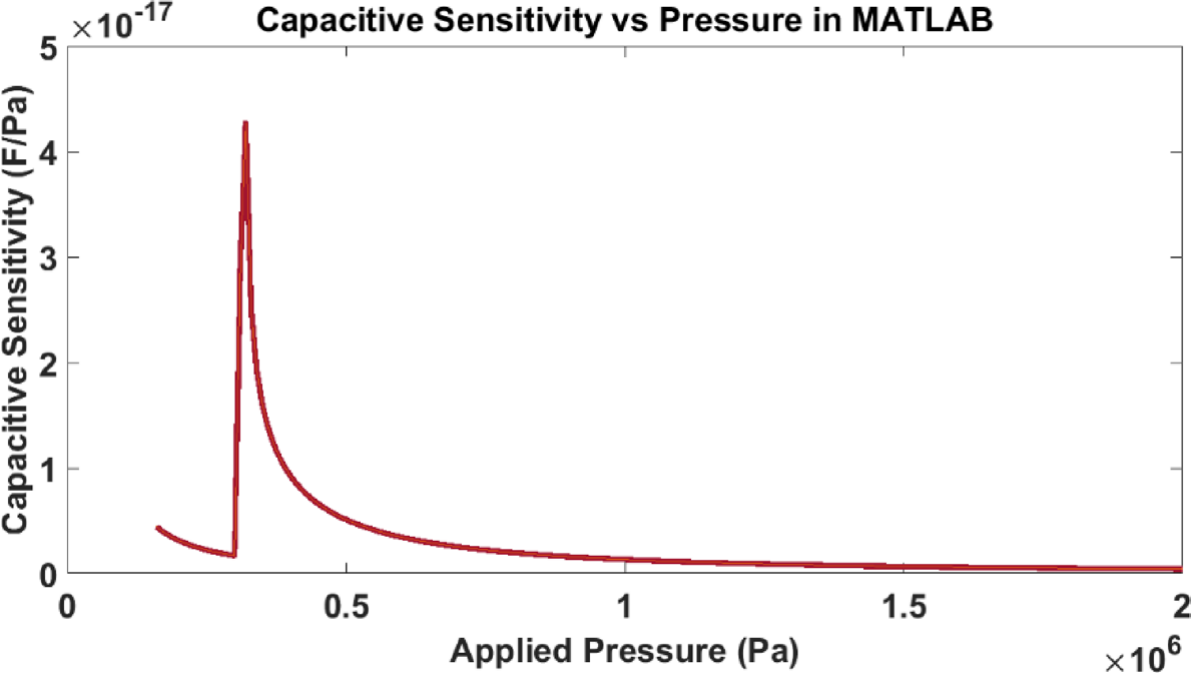
Variation of capacitive sensitivity with pressure.

As shown in Fig. 8 with increasing applied pressure, the capacitive sensitivity progressively declines and reaches a near-constant value beyond 2 MPa. A peak is observed around 0.28–0.3 MPa, marking this transition. Beyond this region, in the touch mode (0.3–1.75 MPa), the sensitivity decreases gradually, as the diaphragm is already in partial contact with the substrate, and further pressure increases primarily expand the contact area. In the saturation mode the sensitivity approaches near-zero values as the diaphragm makes almost complete contact, and additional pressure causes minimal capacitance change.

### Influence of diaphragm thickness on mechanical sensitivity responsiveness (validation of sizing effect)

**Fig. 9:**
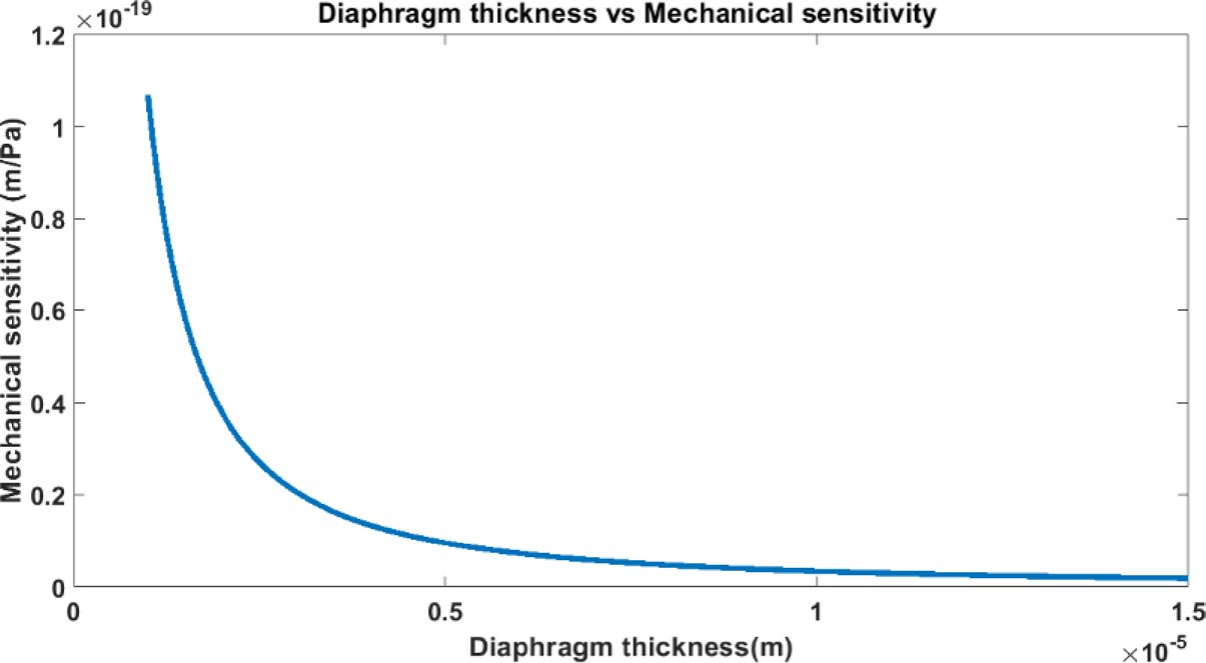
Variation of mechanical sensitivity with diaphragm thickness.

Figure 9 presents the variation of mechanical sensitivity with respect to changes in diaphragm thickness. The results clearly indicate that mechanical sensitivity decreases as the diaphragm thickness increases. This behaviour is attributed to the reduction in diaphragm deflection with increased thickness, due to enhanced structural stiffness. Furthermore, the plot reveals that beyond a thickness of 10 μm**,** any further increase results in a significant drop in mechanical sensitivity. Based on this observation, a diaphragm thickness of 10 μm has been selected as the optimal design constraint for the proposed sensor.

### Comparative analysis with reported works

The comparison presented in Table 2 highlights the superior performance of the proposed wing-shaped dielectric-based CPS in capacitance and linear operating range. With a capacitance value of 15.9 pF, capacitive sensitivity of 0.4 × 10^−16^ F/Pa, and wider linear operation range of 0.3–1.75 MPa, the proposed design significantly outperforms other configurations. The results confirm the robustness and high-pressure range suitability of the proposed sensor design.

**Table 2:** Key Performance Parameters Comparison Between Proposed Sensor and Reported Designs.

Sensor type	Substrate & diaphragm	Size (μm)	Capacitance values obtained in pF	Capacitive Sensitivity in 10^–16^ F/Pa	Linear operation range in MPa
STMCPS^27^	Fixed electrode & Circular	a = 180, h = 5, and g = 2	9.8	0.095	0.2–1.6
STMCPS^28^	Concave well & Circular	a = 180, h = 5, g = 2	3.25	0.021	0.4–1.4
DTMCPS^29^	Convex & Circular	a = 180, h = 5, and g = 2	8.5	0.181	0.2–1.4
DTMCPS^30^	Concave & Circular	a = 180, h = 5, g = 2, and g_n_ = 1	6.2	0.19	0.2–1.1
Proposed work	Wing-shaped dielectric	a = 180, h = 10, and g = 3	15.9	0.4	0.3–1.75

## Fabrication steps

**Fig. 10:**
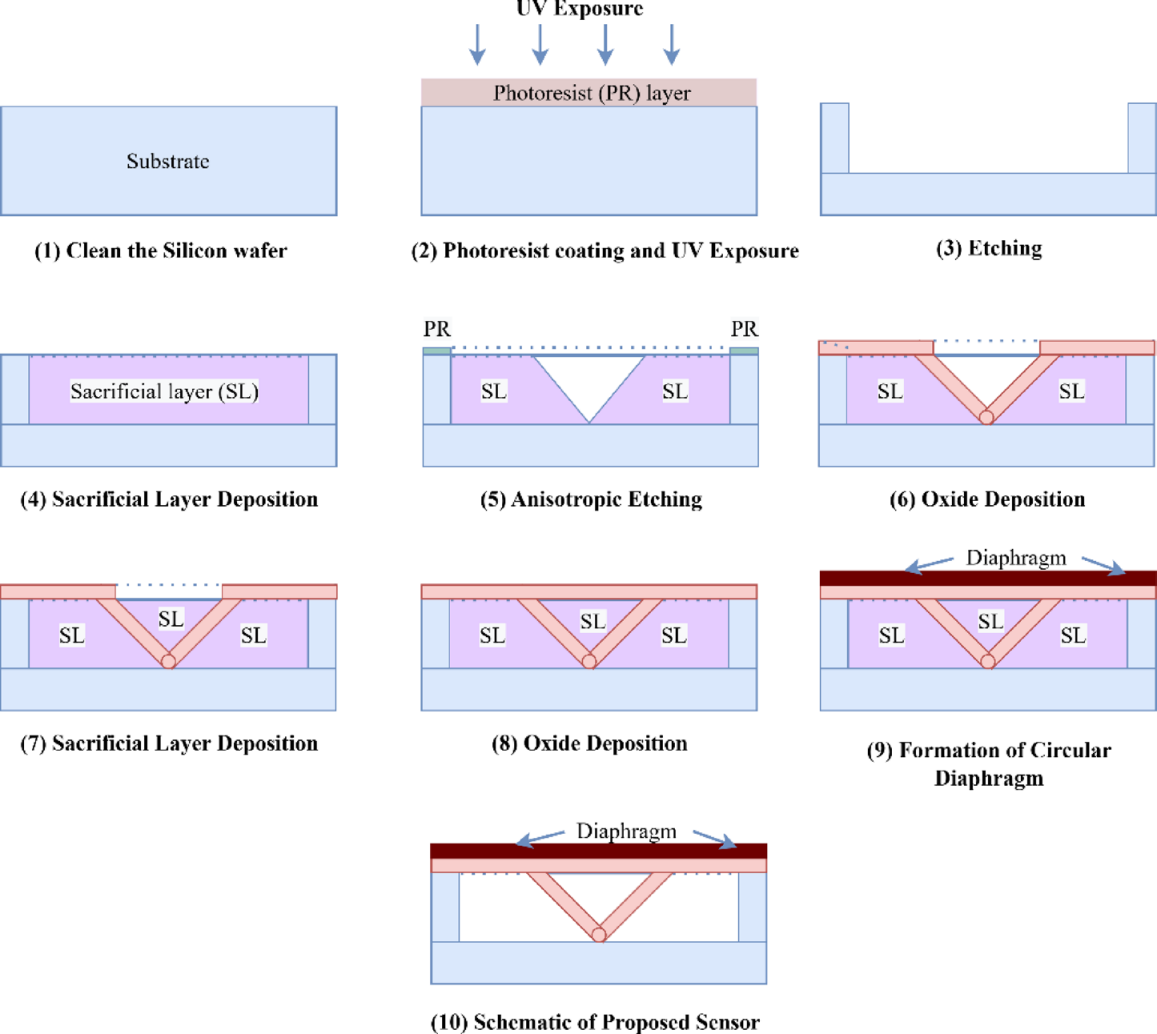
Steps for fabrication.

Figure 10 shows the fabrication steps for the proposed sensor. A clean silicon (Si) substrate serves as the mechanical foundation for the surface micromachining. The substrate is covered with a 2 μm-thick sacrificial layer, typically Silicon dioxide (SiO_2_). A photoresist (PR) layer is then applied and patterned to delineate the slanted trench sections. The sacrificial layer is subsequently shaped into V-groove cavities via anisotropic etching, which will determine the wings’ slant angle of 33.8° and a slant length of 3.6 μm. To provide mechanical support for the wing tips, a tiny anchor point is created at the base of the trench. The dielectric wing structures are then formed by filling the etched gap with 0.5 μm thick Silcion Nitride (Si_3_N_4_) using an uniform deposition process. The diaphragm and wing limits are defined by applying and patterning another layer of photoresist. The wings are then shaped by selectively etching the Si_3_N_4_ layer, and the silicon diaphragm, which is 10 μm thickness, is defined using the proper etching procedures. Lastly, a reactive ion etch procedure is used to remove the sacrificial layer, releasing the Si diaphragm and SiN_4_ wings.

## Conclusion

This study introduces a novel structure to MEMS-based CPS by integrating dual insulating layers on the upper substrate, forming a wing-like structural mechanism that significantly improves responsiveness and sensitivity. The design enables the early formation of a touch point under pressure, reducing the required deflection distance and enhancing sensitivity. The key parameters, such as capacitance variation, capacitive sensitivity, and diaphragm deflection, have been thoroughly analysed to validate the effectiveness of this design, with the accuracy of the findings confirmed through MATLAB simulations and COMSOL Multiphysics validation. The improved capacitive sensitivity of this enhanced CPS design is particularly well-suited for high-pressure applications.

## Data Availability

The authors declare that the data supporting the findings of this study are available within the paper.

## Code availability

Licensed version of MATLAB and COMSOL Multiphysics (Version-COMSOL 6.2 and URL is https://www.istem.gov.in/) software were utilized to generate plots.
